# NIHSS is deficient in acute stroke presenting with cortical deafness; clinical skills remain the backbone: a case report

**DOI:** 10.1186/s41983-023-00645-3

**Published:** 2023-03-27

**Authors:** Tamer Roushdy, Narges W. Mikhail, Shaimaa Ramadan Abdelaziz

**Affiliations:** Neurology Department, Faculty of Medicine, Ain Shams University, 38 Abbasia, PO 11591, Cairo, Egypt

**Keywords:** Cortical deafness, National institutes of health stroke scale, Clinical skills, Thrombolysis, Deficient NIHSS

## Abstract

**Background:**

National institutes of health stroke scale (NIHSS) is used, since its appearance in analysis of stroke in any national or international single center or multicenter study. It is also the golden standard assessment scale for stroke patients whether by emergency medical services on the way to hospital or by emergency room staff and by neurologists whether juniors or senior consultants. Yet, it is not capable of identifying all stroke cases. Along the current case report a relatively rare case of cortical deafness is presented highlighting its rarity and its vascular mechanism as well as how defective is NIHSS in recognizing it.

**Case presentation:**

72-year-old female patient presented with sudden episodic less than 60 min duration of bilateral deafness; initial imaging showed right hemispheric encephalomalacia of old stroke. Patient was initially managed as psychogenic case especially that her NIHSS was zero. On returning again to emergency room she was administered thrombolysis and regained full hearing power. Follow-up imaging revealed a new ischemic stroke along her left auditory cortex explaining her cortical deafness.

**Conclusion:**

Cortical deafness might be missed especially that NIHSS does not detect it. NIHSS role as the only golden standard scale for diagnosing and following up stroke cases ought to be revised.

## Background

The national institutes of health stroke scale (NIHSS) is a valid and a reliable scale that was first introduced in 1983 by the national institute of neurological diseases and stroke (NINDS) and was used in the trial of evaluation of recombinant tissue plasminogen activator (rtPA) [1].

Originally as stated the NIHSS was used in a clinical trial, and since then, it is used repeatedly along different stroke trials and studies whether being conducted on national or international multicentric basis. This could be attributed to its reproducibility, easily performance by any clinical staff whether nurses or doctors and even ability to be applied by emergency medical services (EMS) while on the way to the hospital [2].

The NIHSS is translated and validated to different languages as Chinese, German, Spanish, and Arabic as well as other languages yet without validation and this makes it a universal acceptable scale for stroke assessment at baseline and also in follow-ups [3–6].

It is liable to be performed through telemedicine [7] yet being an observational scale make it not a self-rating scale or telephone performed one and this is well-known among different NIHSS applying staff. Emergency service providers consider NIHSS more than enough, while some neurologists see it as an insufficient scale along a variety of stroke cases [1, 8].

Along the current case report and after an informant written consent, insufficiency of NIHSS is presented in a case of cortical deafness.

## Case presentation

72-year-old female patient, right-handed, with past history of ischemic heart disease and myocardial infarction, old cerebrovascular stroke dating 2006 with no disability on modified Rankin scale (mRS) presented to Emergency room (ER) by recurrent more than 4 episodes of bilateral hearing loss that resolves spontaneously without any deficit within 60 min.

On history taking, there was history suggestive of psychological stress 2 days prior to the patient symptoms onset. Meanwhile, patient denied any previous central or peripheral hearing impairment, traumas, or surgical operations to her head or ears.

NIHSS was performed, while the hearing loss was present, and since the patient could not hear; some remarks were placed beside some domains, and other domains were performed by imitation. The NIHSS score was zero indicating normality for consciousness, commands, gaze, visual field, facial symmetry, motor, coordination, sensory, language, speech, and attention. The only missed to score question was commands about age and month but this was remarked and on writing down these questions; the patient answered loudly.

**Fig. 1:**
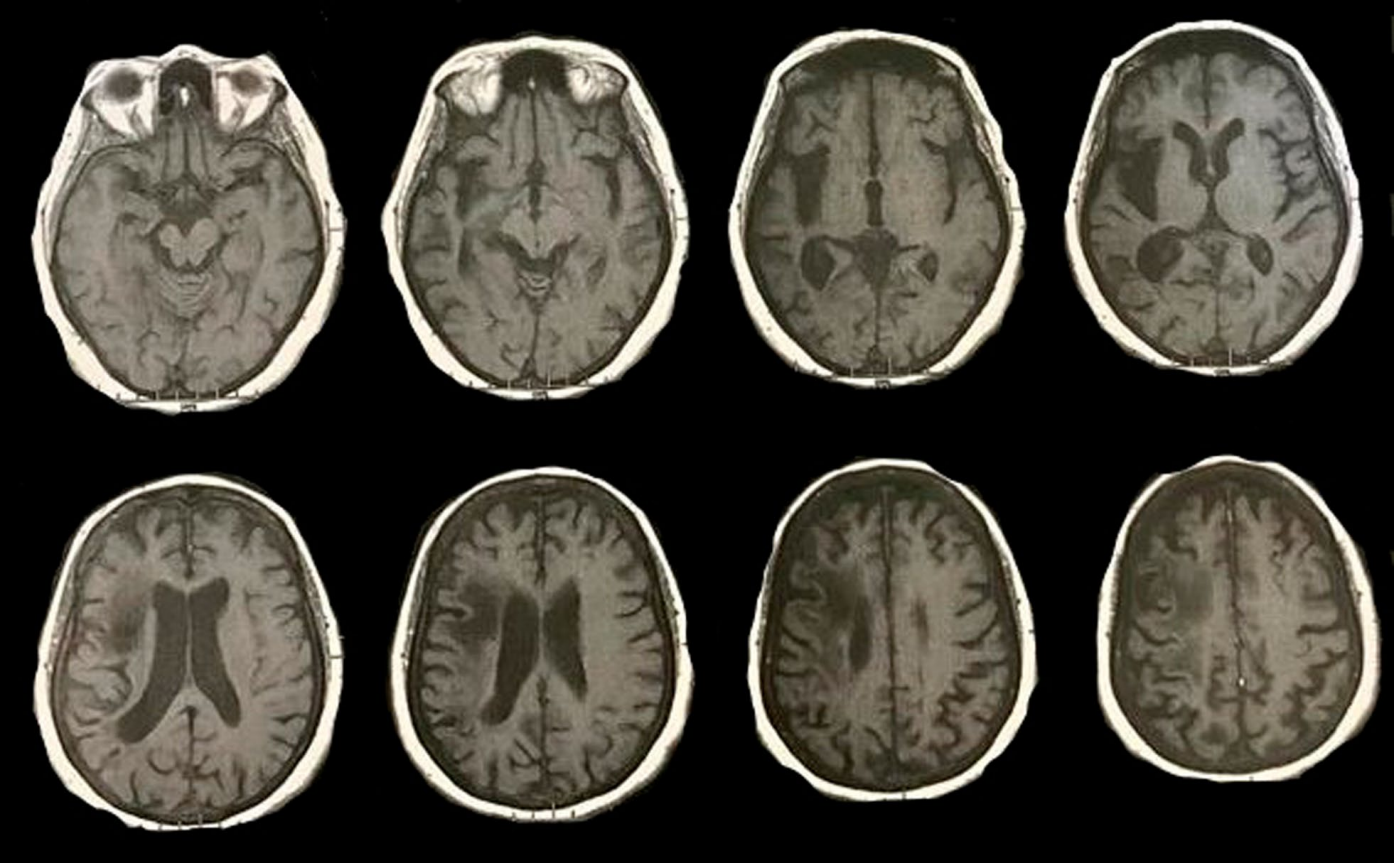
Baseline computed tomography of the presented case, showing encephalomalacia along the right hemisphere with dilated insular ribbon and ipsilateral posterior horn of lateral ventricle

**Fig. 2:**
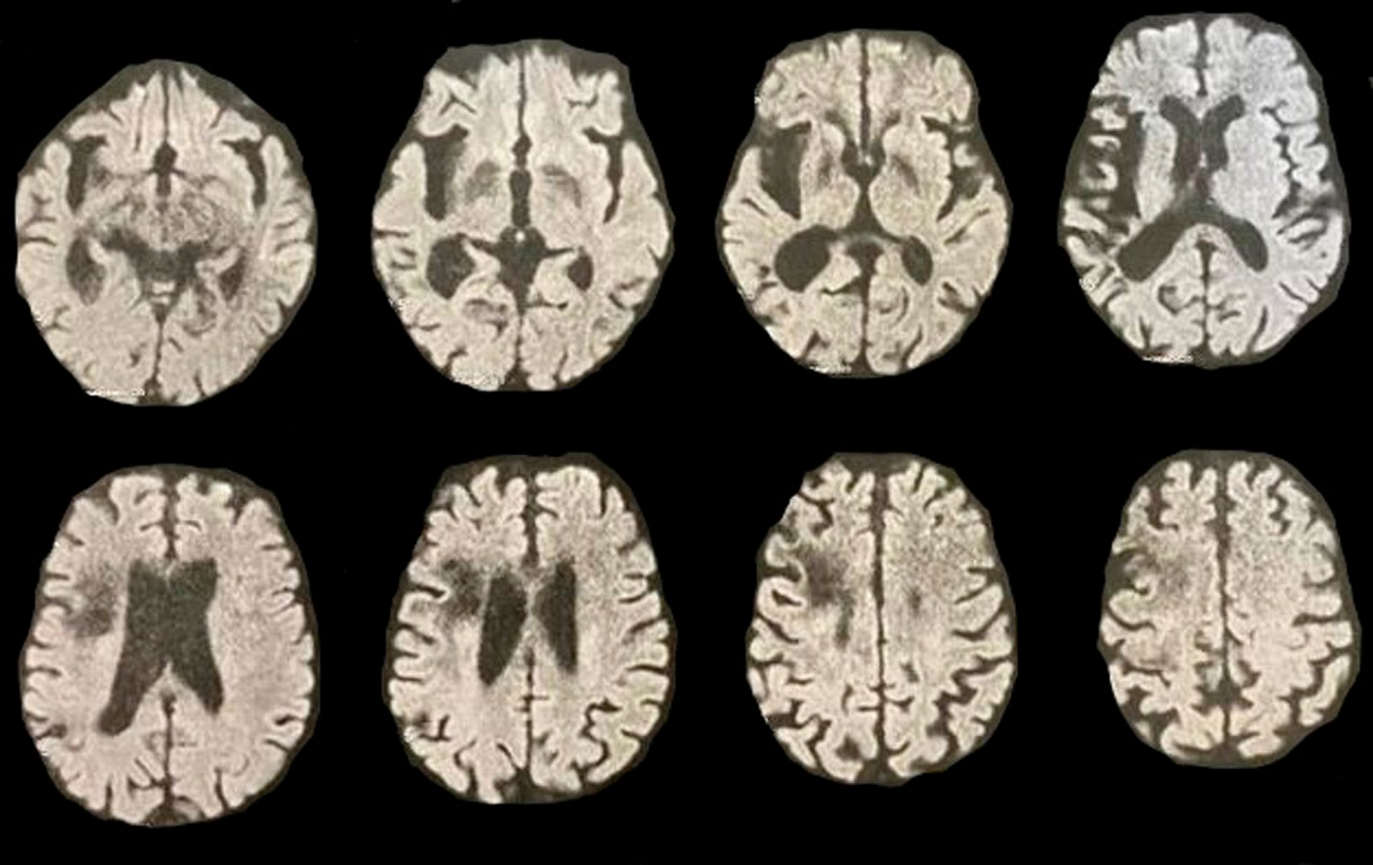
Baseline magnetic resonance imaging diffusion film of the presented case showing dilated right insular ribbon and ipsilateral posterior horn of lateral ventricle donating the patient’s old stroke

Baseline computed tomography (CT) was ordered and it showed dilated right insular ribbon and encephalomalacia along right parietal lobe and dilation of ipsilateral ventricle reflecting the old stroke (Fig. 1). Magnetic resonance imaging (MRI) diffusion (Fig. 2) was ordered that did not add further findings to those of the CT. Patient was reassured that nothing to worry upon and her relatives were informed that her symptoms mostly have a psychogenic background and the patient was asked to follow-up in outpatient clinic.

One hour later; bilateral deafness reoccurred and lasted more than 60 min and patient rushed to ER again.

Based on the facts that psychogenic diagnosis ought to be considered after exclusion of organic causes, patient presentation is sudden and episodic with full resolution in 60 min or less which fulfills the diagnosis of transient ischemic attack (TIA), but now symptoms exceeded the time of just being TIA, the on-call consultant was notified.

The patient has no contraindications to rtPA and even if her deafness was psychogenic in nature; there is no contraindication to administering rtPA in mimics. Thrombolysis decision was taken considering that old infarction in CT could have damaged the ascending ipsilateral auditory pathway and the crossed contralateral ones. The patient condition could be attributed to ischemia involving the auditory cortex on the left hemisphere that if present will affect the still functioning ipsilateral and the crossed contralateral auditory pathways causing the patient complete deafness.

Based on the patient weight; 0.9 mg/kg rtPA was administered, patient received a total of 70 mg. The patient deafness improved on injection and she was transferred to the inpatient to fulfill the stroke protocol including full MRI study.

**Fig. 3:**
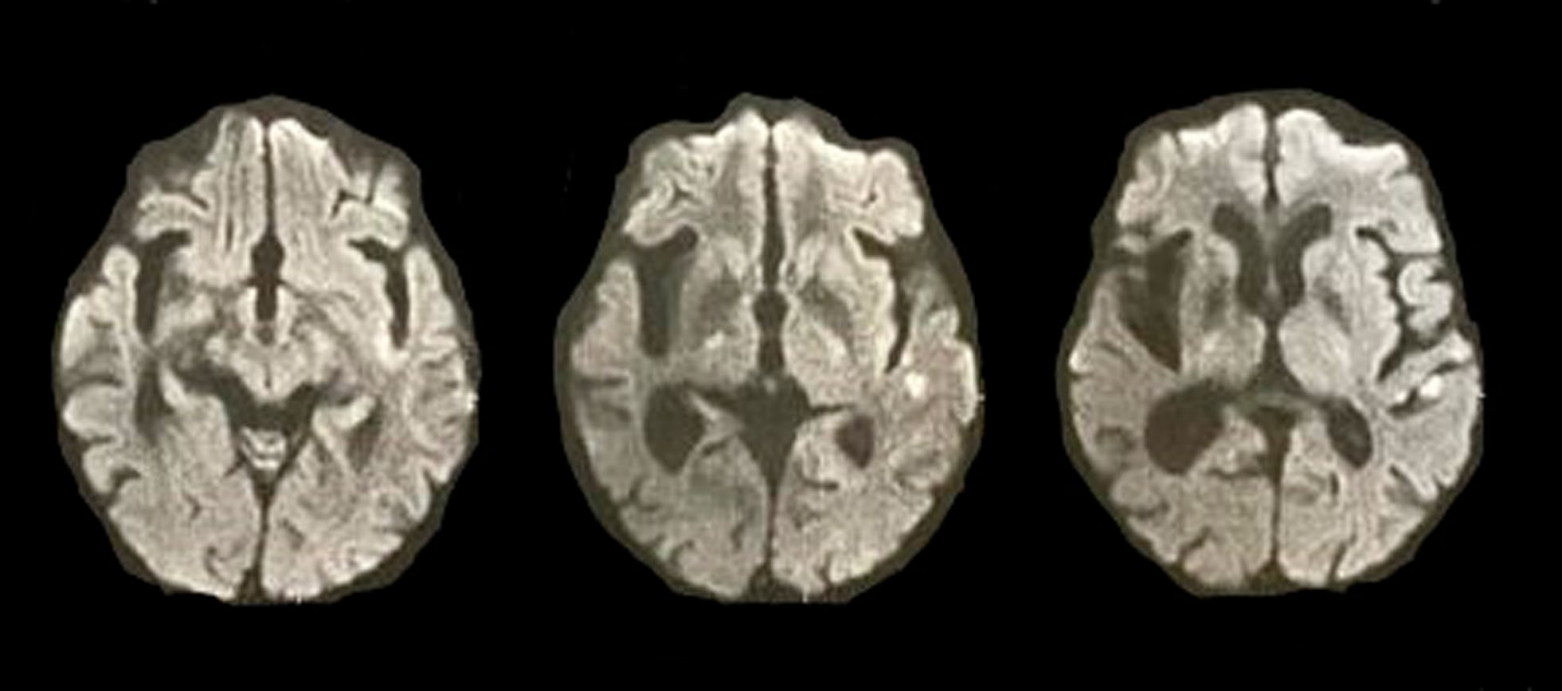
Follow-up 24 h magnetic resonance imaging diffusion film showing restricted vascular lesion along left insula and cortical auditory center

Follow-up MRI after 24 h from injection revealed a lacunar infarction inferior to the left insula corresponding to the auditory cortex (Fig. 3).

Transthoracic ECHO cardiography (Vivid E9 machine, General Electric, Vingmed Ultrasound, Horten, Norway) showed ejection fraction (EF) 61% with normal cardiac chambers dimensions and grade 1 diastolic dysfunction. Transcranial duplex (Siemens, Korea) revealed diffuse atherosclerosis. Patient’s laboratory results were within normal limits. Patient was discharged with mRS zero after 48 h.

## Discussion

The aforementioned case highlights two important points. First it represents a rare case of cortical deafness (CD) and second it shows that NIHSS score might be misleading and even defective in some stroke cases to the extent that if a clinician depends upon it only without clinical skills, imaging, and patient’s comorbid vascular risks, he might miss the case.

CD is a rare neurological disorder. In a systematic review by Silva and colleagues in 2021 a total of only 44 cases were found dating from 1977 to 2021. Along this review a conclusion was made that CD is mostly underdiagnosed as well as being rare and that it develops from cortical and subcortical vascular insults as well as other lesions interfering with the central auditory pathway [9].

Auditory impairment might occur after large right subcortical hemispheric insult. Yet, CD occurs only from bilateral lesions [10]. This was the condition in the presented case; the patient suffered earlier large right subcortical hemispheric infarction with mRS zero and actually there was no recall of initial deficit and on suffering a new left infarction that was presenting initially in the form of TIA a profound CD developed, although patient was still able to pantomime, read orders and write and this by itself might differentiate CD patients from aphasic patients [9].

**Fig. 4:**
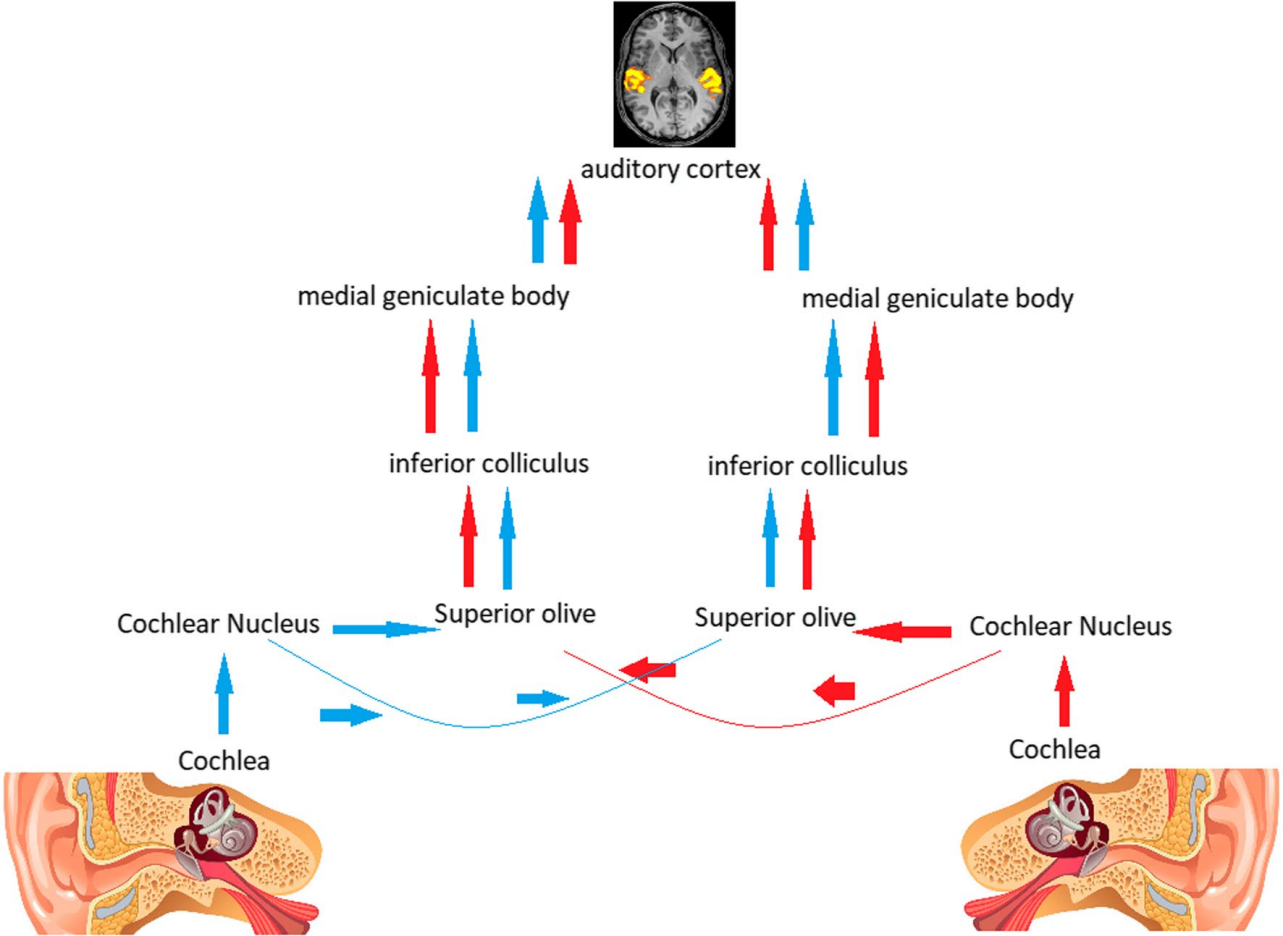
Auditory pathway and how it is bilaterally represented through crossing fibers

Unrecalled earlier stroke might be secondary to that although affecting central auditory pathway unilaterally yet it did not cause profound CD as auditory pathway is bilaterally represented. Sounds are transmitted through the auditory nerve (AN), where it starts the auditory pathway. The AN synapse at the cochlear nucleus. Fibers of the AN cross to the superior olivary complex. Crossing fibers are also detectable along the entire pathway of the auditory system so for CD to occur it needs a bilateral lesion affecting ipsilateral and contralateral fibers of auditory pathway [11] (Fig. 4).

Meanwhile, the patient’s NIHSS was entirely normal. Instructions for applying NIHSS include writing a remark beside points the patient is unable to perform in case of language barrier or any local cause-like fracture or amputation as well as use of imitation and pantomiming in certain occasions [12, 13].

Although widely accepted as a standard scale for stroke assessment, diagnosis, and prognosis [14] yet, NIHSS has drawbacks. In general, it is directed to anterior circulation strokes rather than posterior ones. NIHSS focus more on anterior left hemispheric strokes rather than right ones; its items of verbal commands justify scoring a deficit in case of aphasia, and it has a domain for language. NIHSS in aphasia might score 7 points more if compared to scoring 2 points in neglect. As a result, patients with left hemisphere stroke with aphasia usually score higher scores [15].

NIHSS even may score minimal values and the patient still have a large vessel occlusion (LVO) as those of the RESCUE Japan registry-2 [16] and if imaging modalities are not available as in-case of absence of cerebral angiography or magnetic resonance angiography patients might not be offered the opportunity for rtPA or thrombectomy especially in countries, where resources are minimal and in the current economic crisis and delay in chain of supply after COVID-19.

It is worth mentioning that although NIHSS has an item for assessing visual field yet, as cortical deafness; field affection secondary to optic pathway insult is often missed and many cases are accidently encountered with field defect only in case of suffering a new insult presenting with motor deficit which is much relied upon in NIHSS. This was the core of Falkenberg et al.’s study when used the term “Invisible” reflecting how patients and health care providers might miss visual impairment as a cause of stroke [17].

Our presenting case that highlights how NIHSS is defective in detecting acute vascular hearing impairment agrees with a systematic review by Hanna et al., where they concluded that scales used for stroke assessment including NIHSS are deficient in fully analyzing special senses like vision and that other tools ought to be invented [18].

In the current case, NIHSS failed to recognize the patient’s central vascular deficit to the extent that provisionally her condition was considered psychogenic and she was dismissed from ER to follow-up in outpatient clinic. Only after her second return rtPA was considered but again on a zero NIHSS score and after analyzing the patient’s entire available data including imaging. Acute CD and as a result of missed identification by NIHSS sometimes may be considered psychiatric [9].

Unlike cases discussed by Silva and colleagues that showed improvement to an extent in some cases to reach pure word deafness, while most of cases did not improve, the current case improved markedly on receiving rtPA.

## Conclusions

The current case report presents a rare and occasionally missed identified condition which is cortical deafness and add to the increasing evidence that widen the gap between neurologists and ER specialists about crucial defects in NIHSS in some cases that might extend to missing rtPA administration owing to normal NIHSS scores for some cases despite arriving to ER in window.

Reconsidering NIHSS value is a must, validating and using additional scales whether related to NIHSS as the expanded NIHSS (eNIHSS) or other scales is to be considered, clinical skills still matter.

## Data Availability

The corresponding author takes full responsibility for the data, has full access to all of the data, and has the right to publish any and all data separate and apart from any sponsor.

## Abbreviations

NIHSS: National institutes of health stroke scale
rtPA: Recombinant tissue plasminogen activator
EMS: Emergency medical services
mRS: Modified Rankin scale
ER: Emergency room
CT: Computed tomography
MRI: Magnetic resonance imaging
TIA: Transient ischemic attack
EF: Ejection fraction
CD: Cortical deafness
AN: Auditory nerve
LVO: Large vessel occlusion
eNIHSS: Expanded national institutes of health stroke scale
